# Research progress of bone metastases: From disease recognition to clinical practice

**DOI:** 10.3389/fonc.2022.1105745

**Published:** 2023-01-25

**Authors:** Wenbo Yang, Qing Pan, Fuhua Huang, Hongzhi Hu, Zengwu Shao

**Affiliations:** Department of Orthopaedics, Union Hospital, Tongji Medical College, Huazhong University of Science and Technology, Wuhan, China

**Keywords:** bone metastases, disease recognition, clinical practice, review, scope

## Abstract

Bone metastases, as one of the common types of metastatic tumors, have a great impact on the survival period and quality of life of patients. Bone metastases are usually characterized by bone destruction. Skeletal related events caused by bone destruction often lead to pain, pathological fractures and even paralysis. In this review, we provide a detailed explanation of bone metastases from the epidemiology, clinical features, pathogenesis, and recently developed clinical treatment viewpoints. We concluded that the incidence of bone metastases is increasing gradually, with serious clinical symptoms, complex pathogenesis and diverse clinical treatment. Tumor cells, immune cells, osteoblasts/osteoclasts and other cells as well as cytokines and enzymes all play a key role in the pathogenesis of bone metastases. We believe that the future treatment of bone metastases will be diversified and comprehensive. Some advanced technologies, such as nanomedicine, could be used for treatment, but this depends on understanding how disease occurs. With the development of treatment, the survival time and quality of life of patients will be improved.

## 1 Introduction

Bone metastases are malignant tumors that colonize bone through such as hematogenous metastases to form bone lesions (1). They are a common complication of many malignant tumors and may lead to poor prognosis (2). As a kind of disease, the epidemiological and pathological features of bone metastases are more complex than those of other malignant tumors. With the development of comprehensive tumor therapy, the survival time of tumor patients has been extended, and the occurrence probability of bone metastases has also shown an increasing trend (3). Once a patient is diagnosed with bone metastasis of malignant tumor, the prognosis will be significantly worse and the quality of life will be significantly decreased. Related complications will significantly increase the financial burden of patients and families (4). Therefore, bone metastases of malignant tumors have gradually attracted extensive attention from clinicians and clinical researchers. With the development of relevant scientific experiments and clinical studies, clinicians’ views on the treatment of bone metastases are constantly being updated, from the previous negative conservative treatment and analgesic treatment to the current personalized comprehensive treatment such as surgery, radiotherapy, chemotherapy and targeted therapy, which improves the quality and survival time of patients (5). Multidisciplinary cooperation has also helped improve the quality of life of patients with bone metastases. A variety of medical concomitant symptoms and drug side effects can be diagnosed and treated in time. In addition, based on the development of scientific research in related fields, some newer fields, such as the diagnosis and treatment of bone metastases with nanomaterials, are developing rapidly. In view of the important role of bone metastases in bone and soft tissue tumors, we reviewed the epidemiology, pathogenesis and clinical treatment of bone metastases in order to provide necessary guidance for the development of related disciplines.

## 2 Epidemiological, pathological and clinical features of bone metastases

According to the existing epidemiological data, bone metastases can appear in many types of malignant tumors, especially breast cancer, lung cancer, prostate cancer, kidney cancer and thyroid cancer (6). Bone metastases have been reported in 40% of non-small cell lung cancer (7). More than 70-85% of patients with advanced prostate cancer develop bone metastases (8). The incidence of bone metastases in differentiated thyroid carcinoma(DTC) ranges from 1% to 20%. About 44% of metastatic DTC patients have lesion that has spread to bone (9). About 75% of patients with advanced breast cancer develop bone metastases (10). Bone metastases have been reported in 30% of patients with renal cell carcinoma(RCC) (11). Some reports concluded that bone is the second most common site of RCC metastasis (12, 13). About 35-40% of patients with RCC metastases are bone related (12). In addition, cancers such as bronchial carcinoma often cause bone metastases (14). The incidence of bone metastases in gastrointestinal cancer is relatively low (14). According to current clinical observation, it is rare for gastrointestinal tumors to develop bone metastases without liver and lung metastases. The relevant data is described in **Table 1**. Clarifying the relevant epidemiological data of bone metastases has important clinical significance, which can help clinicians to make a comprehensive assessment of patients with related malignant tumors and develop appropriate follow-up protocols.

**Table 1:** A summary of the types of bone destruction, occurrence probability and common sites of bone metastasis.

Type of primary tumor	Main type of bone destruction	Proportion of metastasis	Common site of metastasis
Lung cancer	Osteolytic destruction	40%	Spinal metastases: ——thoracic vertebra (70%) ——lumbar vertebra (20%) ——Cervical and sacral vertebrae (10%) Pelvis; Femur: ——Neck of the femur (50%) ——Subtrochanteric site (30%) ——Intertrochanteric site (20%); Other parts of long bones.
Prostatic cancer	Osteogenic destruction	>70-85%	
Breast cancer	Osteolytic destruction	75%	
Thyroid cancer	Osteolytic destruction	1~ 20%	
Renal carcinoma	Osteolytic destruction	30%	

The concluded data presented in the table are reported in partial typical literature. Relevant references are reflected in the preceding paragraphs.

The pathological features of bone metastases are also varied. For common bone metastases, some bones show a higher incidence, such as the spine, pelvis, femur, humerus and so on (15). Studies have indicated that spinal metastases are common in patients with advanced malignancies, with a reported incidence of 30-50% (16, 17). The prevalence of spinal metastases in some malignancies may even be as high as 70%, with most metastases occurring in the thoracic spine (70%), followed by the lumbar spine (20%) and the cervical and sacral vertebrae (10%) (18, 19). Some long bones such as humerus and femur may also exhibit bone metastasis. Guzik noted in his study that about 10% of patients with primary malignant tumors develop proximal femur metastases (20). In metastatic tumors of the femur, 50% of the lesions occurred in the neck of the femur, 30% occurred in the subtrochanteric site, and 20% occurred in the intertrochanteric site, which may be related to the local developed blood supply (20). Wedin et al. mentioned in their study that the humerus is the second most common site of bone metastases in long bones (21). The common metastatic sites showed more cancellous bone and more abundant blood circulation. In addition, different bone metastases have different forms of bone damage. According to the changes of bone content in the lesion, bone metastases are mainly divided into osteoblastic lesions and osteolytic lesions (22). However, in some patients with bone metastases, both lesions may be present (23). Osteoblastic bone metastases are more common in prostate cancer (24, 25). On the contrary, most of the bone metastases of breast cancer, kidney cancer and other malignant tumors are often presented as osteolytic lesions (26–29). Bone metastases of lung cancer and thyroid cancer are also often presented as osteolytic lesions (30, 31). It is important to identify the relevant pathological mechanism for subsequent treatment. The clinician can give appropriate clinical examination and symptomatic treatment according to the type and location of lesions that may occur. At the same time, the specific mechanism of the lesion is also the key basis for the design of the treatment of bone metastases.

Once bone metastases occur in malignant tumors, they often present complicated symptoms, and the prognosis of patients is often significantly worse. For example, bone metastasis is the main cause of death in prostate cancer patients, and there is no good treatment plan at present (32, 33). When bone metastases occur in patients with DTC, the survival rate may be reduced by more than 60% (34). Patients with bone metastases often experience pain, spinal cord compression, pathological fractures, and bone radiation; these symptoms are known as skeletal-related events (SREs) (35, 36). SREs occurs in a large number of patients with metastatic bone tumors, and brings great difficulties to the treatment. For example, it has been reported that 30-40% of patients with advanced lung cancer develop bone metastases that lead to SREs, which causing hypercalcemia, pathological fractures, spinal compression, and bone pain, leading to a poor prognosis (14, 37). SREs associated with bone metastases in prostate cancer have also been reported (38). Although bone metastases of prostate cancer are mainly osteoblasts, pathological fractures are still common (39). This may be due to the fact that the mechanical properties and structure of the diseased area are abnormal despite the “bone formation”. Liu et al. mentioned in their study that more than 70-85% of patients with advanced prostate cancer develop bone metastases, which are characterized by severe pain and an increased possibility of fracture (8). Bone metastases with SREs have been reported to induce lower survival rates (40). After metastatic renal carcinoma metastases to the spine, pelvis and proximal femur, SREs such as pain, pathological fracture, hypercalcemia and spinal cord compression may occur, seriously affecting the quality and survival time of patients (12). Particularly severe SREs include pathological fractures, spinal cord compression and hypercalcemia requiring dialysis, which can incapacitate the patient in a relatively short period of time, and can quickly become life-threatening. In addition, due to the comprehensive impact of SREs on patients with bone metastases, the overall health of patients may deteriorate rapidly in a short time, making it difficult for them to withstand radiotherapy and chemotherapy with greater side effects. Therefore, clinicians should detect, diagnose and treat patients with bone metastases as early as possible, and take necessary preventive measures for possible serious complications. Laboratory and imaging tests such as X-ray, CT, MRI, bone scans, and tumor markers should be considered and used if necessary to achieve early and accurate diagnosis.

## 3 Advances in the pathogenesis of bone metastases

**Figure 1:**
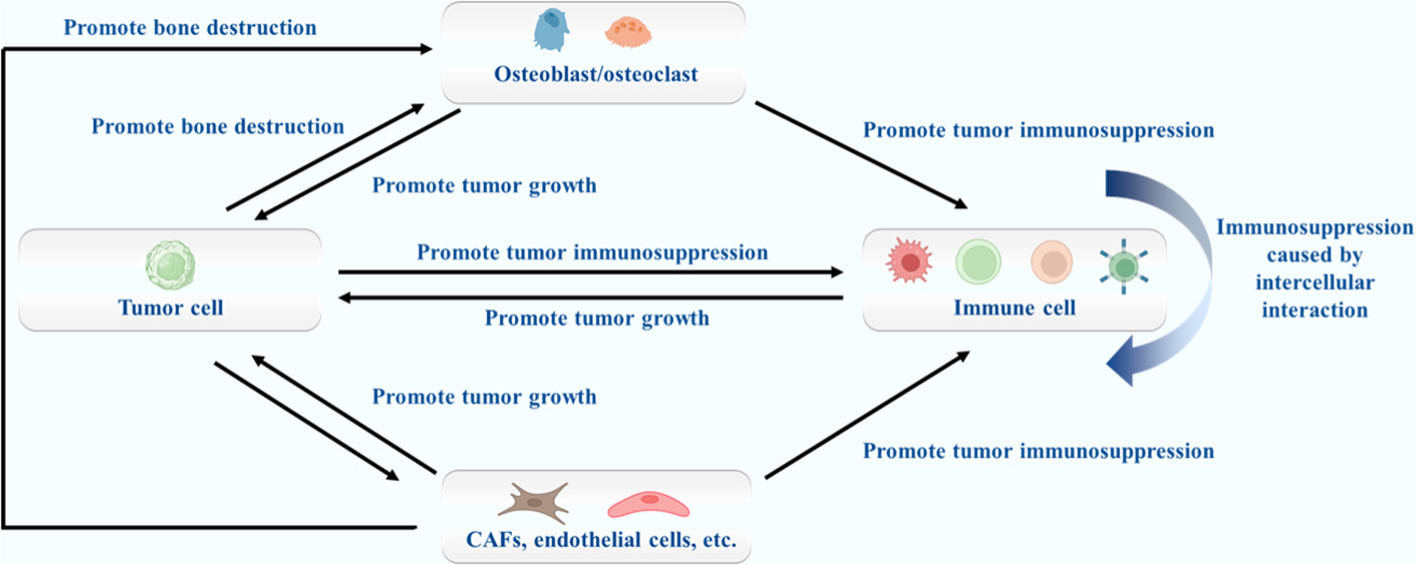
The role of different cell types in bone metastases.

At present, studies on the pathogenesis of bone metastases are increasing, including the formation mechanism of bone metastases and the pathogenesis of local bone destruction. These scientific studies provide an important reference for clarifying the pathophysiology and clinical treatment of diseases. From the perspective of pathophysiology, bone metastases are a comprehensive disease. Tumor cells, osteoblasts/osteoclasts, immune cells and other components all play an important role in the pathogenic process. The relevant contents are shown in **Figure 1**. The pathogenesis and development of bone metastases will be discussed from the perspectives of tumor cells, osteoblasts/osteoclasts, immune cells, cytokines and other possible aspects.

### 3.1 The role of tumor cells in the pathogenesis of bone metastases

Metastatic tumor cells are one of the major players in bone metastases. In essence, the occurrence of bone metastases is a coordinated process in which malignant tumor cells leave the primary site to spread to bone and survive in the bone microenvironment (41). The metastases of malignant tumors generally include tumor cells leaving the primary site, entering the blood circulation and ectopic colonization. In particular, for bone metastases, circulating tumor cells reside and become dormant in the normal vascular of the bone marrow long before clinically detectable metastases develop. Over time they proliferate and regulate the function of bone resorption (osteoclasts) and bone formation (osteoblasts) cells, leading to the development of bone metastases (42). Tumor cells show a tendency to metastasize more easily under certain conditions. For example, the tumor has gene mutation, epithelial-mesenchymal transformation, and metabolic changes. The gene mutation of malignant tumor cells plays a key role in bone metastasis. In a study by Huang et al. on the mechanism of bone metastasis in lung cancer, 425 and 422 genomic alterations were detected in primary and metastatic lesions respectively (43). There were significant differences in tumor mutational burden between primary lung adenocarcinoma and matched bone metastases (43). This indicates that tumor mutational burden may play a role in bone metastasis of lung cancer. Arnold et al. mentioned in their study that the number of somatic mutations in the site of bone metastasis was statistically significantly higher than that in the site of primary or soft tissue metastasis (44). Bartels et al. concluded through their study that mutations in ESR1 are associated with estrogen receptor expression as well as high proliferative activity, and affect bone metastases in a part of estrogen receptor positive breast cancers (45). However, the current researches on the influence of gene mutation on the occurrence of bone metastases are mostly reflected in the research level of epidemiological data statistics, and there are few in-depth researches on the mechanism. In the future, related research needs to be further in-depth. In addition to some reported gene mutations, epithelial mesenchymal transformation (EMT) in tumor cells may also be an important factor promoting the occurrence of bone metastases. EMT refers to the differentiation and transformation process of epithelial cells into mesenchymal cells, which is believed to be related to tumor progression including tumor metastasis (46, 47). Several studies have been published on the pathogenesis of the relationship between epithelial mesenchymal transformation and bone metastases. Liu et al. pointed out in their study that Notch3 was associated with EMT and overexpressed in bone metastases of NSCLC, and inhibition of Notch3 expression could reduce the invasion ability of NSCLC cells *in vitro (* 48). Epithelial mesenchymal transformation may also be associated with metastasis of malignant tumors such as breast cancer and prostate cancer. Horas et al. confirmed that the deficiency of vitamin D receptor(VDR) in human breast cancer cells can promote can promote EMT and the spread of cancer cells (49). In the study of the pathogenesis of bone metastases, EMT is often mentioned (50, 51). Therefore, EMT can be used as a key breakthrough in future research on the treatment of bone metastases. In addition, metabolic changes of tumor cells are also considered to be a key factor in the development of tumor metastasis (52). Studies have shown that different tumor stem cells adapt to unique metabolic characteristics for organ metastasis (53). Thysell et al. analyzed the metabolism of bone metastasis in prostate cancer and identified metabolites such as cholesterol that might be associated with prostate cancer metastasis (54). In addition to the mechanisms mentioned above, cancer stem cells (CSCs), a new concept proposed in recent years, are also believed to be closely related to bone metastasis of tumors (55). Based on existing studies, we believe that in bone metastases of malignant tumors, the tumor cells should usually be changed compared to the primary site. Such changes may be at the genetic level, at the metabolic level, or at the cellular phenotype level. However, the specific changes of bone metastases in different malignancies may be different, so specific studies are needed. At present, there is still a relative lack of research on mutant genes or altered metabolic functions. After the relevant epidemiological data are revealed, more mechanism studies should be conducted to identify the target of bone metastasis and design corresponding interventions.

### 3.2 The role of osteoblasts/osteoclasts in the pathogenesis of bone metastases

**Figure 2:**
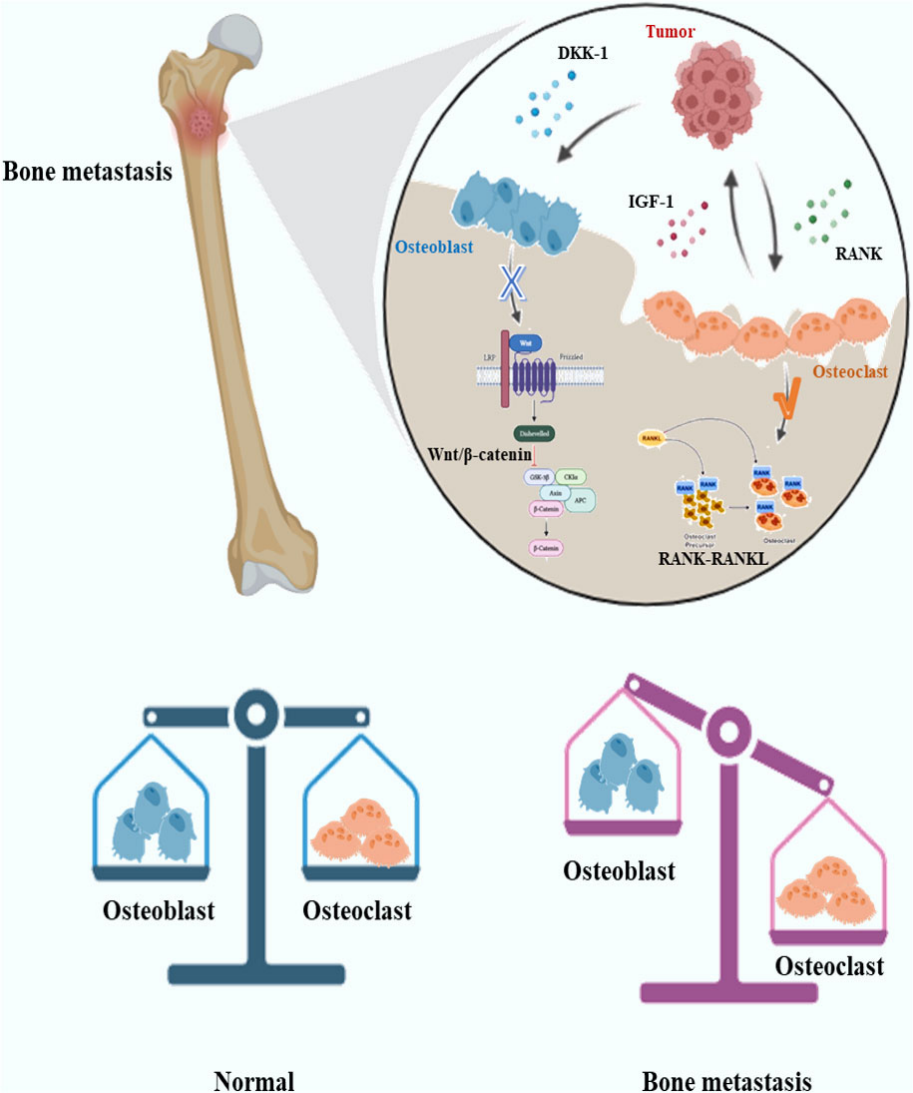
Schematic diagram of the interaction between tumor cells and osteoblasts/osteoclasts in osteolytic bone metastases. In osteolytic lesions, tumor cells may secrete molecules such as DKK-1 to inhibit osteoblast Wnt signaling pathway and promote osteoclast function through RANK-RANKL signaling pathway. Osteoclasts may secrete IGF-1 and other molecules to promote tumor growth.DKK-1, Dickkopf-1; IFG-1, Insulin-like growth factor 1; RANK, Receptor Activator of NF-κB.

The role of osteoblasts/osteoclasts in bone metastases has been studied for a long time, and many drugs are gradually being completed in clinical trials. During the occurrence and development of bone metastases, many pathological changes are related to abnormal regulation of osteoblasts and osteoclasts. Inhibition of osteoblasts and abnormal activation of osteoclasts are often the key mechanisms of osteolytic metastases. Osteoblasts and osteoclasts are the direct “executors” of bone destruction in bone metastases, and their regulation may be related to a variety of cells and factors, such as tumor cells, immune cells and inflammatory factors (56–58). For osteoblasts and osteoclasts themselves, Wnt/β-catenin pathway, RANK-RANKL pathway and other pathways closely related to osteogenesis/osteoclast process are the focus of research (59, 60). Wnt signaling pathway may play an important role in bone metastasis of malignant tumors (61). The Wnt pathway and the role of osteoblasts have attracted much attention since bone metastases of prostate cancer are often manifested as osteoblastic lesions. Dai et al. showed in their study that prostate cancer can promote osteoblast differentiation through classical and non-classical Wnt signaling pathways and stimulate BMP-dependent and BMP-independent osteoblast differentiation (62). However, there are some different studies. Aufderklamm et al. have shown that DKK-1, an inhibitor of the Wnt pathway, mediated osteoblast inhibition contributes to prostate cancer progression (63). The RANK/RANKL signaling pathway has also received attention in bone metastases. This pathway mainly affects the function of osteoclasts in the local microenvironment of bone metastases. It has been suggested that the RANK/RANKL signaling pathway is involved in the castration-insensitive prostate cancer (64). SREs can be prevented with the RANKL inhibitor Denosumab (65). Interestingly, RANKL connects bone to the immune system, while RANK-RANKL is a regulator of osteoclast development, lymph node development, bone metabolism, and T cell/dendritic cell communication (66). This suggests that the regulation of the RANK/RANKL signaling pathway does not only affect osteoclasts. Not only the above common pathways, but also the effects of other factors on osteoblasts/osteoclasts have been extensively studied. For example, osteoblasts may be negatively regulated by cancer cells and appear apoptosis (67). The main mechanisms of interaction and regulation of osteoblasts/osteoclasts with tumor cells in osteolytic bone metastases are summarized in **Figure 2**. In the future, some more detailed cell interactions on osteoblasts/osteoclasts in bone metastases should be further investigated, for example, the regulation of osteoblasts/osteoclasts by exosomes produced by bone metastatic tumor cells. RANK/RANKL and Wnt/β-catenin pathways are both downstream signaling pathways. In bone metastatic cancer, which signaling pathway changes may trigger the changes of the above downstream pathways is a more valuable research direction.

### 3.3 The regulatory role of cytokines other key proteins (enzymes)

Different from cells, cytokines are a class of small molecules that regulate cell function with a wide range of effects. Common cytokines include interleukin(IL), tumor necrosis factor(TNF), and so on (68). In the past few decades, cytokines and cytokine receptors have been extensively studied as targets for cancer treatment (69). In the pathogenesis of metastatic tumor, cytokines may be secreted by tumor cells and immune cells, and the target may include tumor cells, immune cells, osteoblasts/osteoclasts, etc. There are many types of interleukin, which is closely related to inflammation and tumor growth, etc. At present, certain studies have been conducted in bone metastases. Claudia et al. reported in their review that IL-1B is important in the inflammatory process, and influences the growth of bone metastases in breast cancer, including angiogenesis, etc. (10). IL-6 overexpression is also associated with bone metastases (70). Interleukin is also produced by osteoclasts to regulate tumor cells. The study of He et al. showed that lung cancer cells induced osteoclasts to secrete IL-19 to act on IL20RB on the surface of lung cancer cells, thus promoting the proliferation and bone metastasis of lung cancer cells (71). Tumor necrosis factor also plays an important role in the development of bone metastases. Hamaguchi et al. found that inhibition of TNF-α has a novel role in reducing or preventing bone metastasis in breast cancer models (72). Interferon has been less studied in bone metastases. Chemokines are a class of cytokines secreted by cells, which can induce the directed migration of nearby cells (73). Chemokines play an important role in metastatic tumors because they have an important effect on cell migration, colonization and other processes. Chemokine/chemokine receptor CXCL12/CXCR4 pathway and CCR3/CCL7 pathway can be used as mediators in the process of bone metastasis and may affect the colonization of tumor cells in bone (74, 75). According to current studies, the interleukin family and chemokine family related pathways may be relatively important in the influence of bone metastases. The design of relevant targeted drugs for these two pathways may be an important idea to delay the progression of bone metastases or prevent the appearance of bone metastases.

In addition to common cytokines some enzymes can also promote the disease progression of bone metastases by influencing immunity and bone formation. Matrix metalloproteinases (MMPs) is a type of enzyme containing zinc, which can decompose extracellular matrix (76). Since MMPs is closely related to the synthesis of bone matrix and the regulation of osteoblasts/osteoclasts, it is believed that MMPs may promote the onset of bone metastases. Pego et al. mentioned in their review that MMPs, especially MMP-9, played an important role in bone metastasis of prostate cancer (77). MMP-9 is also significant in the occurrence and development of other bone metastases, such as breast cancer bone metastases, and may be a therapeutic target for bone metastases (78). In addition, MMPs such as MMP-13 also play a role in promoting bone metastasis of malignant tumors (79). In addition to MMPs, the role of Cyclooxygenase-2 (COX-2) in bone metastases is also attracting increasing attention. COX-2 is a key rate-limiting enzyme in the synthesis of prostaglandin E2 (PGE2), which is closely related to inflammation, tumor growth, angiogenesis and other aspects (80). Studies have shown that COX-2 can increase the proportion of osteoclast and is one of the key genes in breast cancer bone metastasis (81). Karavitis et al. mentioned that COX-2 and PGE2 can regulate bone metastasis by influencing immunity (82). In addition, enzymes such as Indoleamine 2, 3-dioxygenase 1 (IDO1) have also been found to be associated with bone metastases (83). In the future, more enzymes with the potential function of promoting tumor bone metastasis can be identified through RNA sequencing and proteomics. As a special catalyst, enzymes often correspond to certain characteristics of substrates and products, as well as related chemical reactions, which may provide conditions for targeted therapy of bone metastases.

### 3.4 The role of immune cells in the pathogenesis of bone metastases

**Figure 3:**
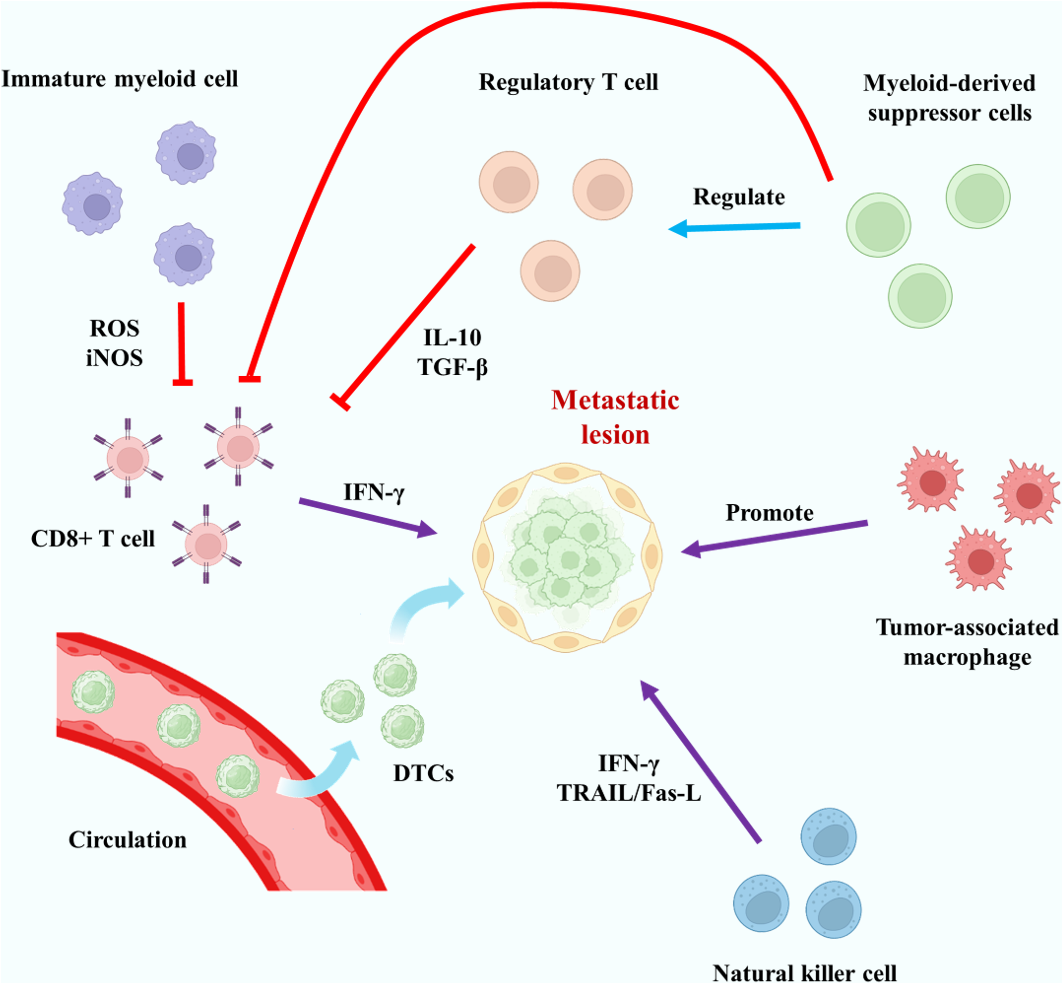
Typical mechanisms of action of key specific and non-specific immune cells in bone metastases. The red lines represent inhibitory effects.

The immune cells in the body include specific immune cells and non-specific immune cells. The specific immune cells include T cells, B cells and so on, and their mechanism of action is often highly specific. Non-specific immune cells include monocytes/macrophages, dendritic cells, etc., which usually exhibit low specificity and are responsible for assisting specific immune cells in some cases. In cancer patients, it is generally believed that local immunity plays a potential role in promoting the occurrence, development and metastasis of tumors. The relevant immune cells may “migrate” to the tumor tissue and “protect” it instead. Interestingly, bone is actually an important immune organ in the body, because bone marrow is an important site of white blood cell production (84). So there has to be a special environment for immune activation to be suppressed. The relationship between bone marrow and metastatic tumors began to be studied earlier, and many cells were found to be related to immunosuppression, such as myeloid-derived suppressor cells (MDSCs) and Mesenchymal stem cells (MSCs) (85, 86). However, the mechanisms related to immune microenvironment are. For example, regarding the Irf7 pathway, existing studies have shown that its role in bone metastasis of breast cancer and prostate cancer seems to be suppressive (87, 88). The immunosuppressive mechanism of bone metastases with different primary lesions should be studied independently. Regulatory T cells (Tregs) play an important role in specific immunity (89). CD4+CD25+ Tregs are an important group of T cells in bone marrow and may be highly related to immunosuppression (90). The study of Tan et al. indicated that the RANKL-RANK pathway may affect the content of Tregs, thus affecting local immunity (91). Tregs can secrete anti-inflammatory cytokines such as IL-10, TGF-β and IL-35, and act on such as CD8+T cells to achieve immune suppression (92, 93). These related cytokines may play a key role in circulating tumor cell dormancy in bone metastases or in tumor cell proliferation in metastatic sites. CD8+T cells are also regulated by immature myeloid cells and osteoblasts (94). In non-specific immunity, macrophages, especially tumor-associated macrophages(TAMs), have a great influence on the pathogenesis of metastatic tumors (95). The role of macrophages is diverse, and under different circumstances they will polarize into different subtypes, mainly including M1 type and M2 type (96). Their main effects on tumor cells are almost opposite, with M1-type macrophages often showing killing effect on tumor, while M2-type macrophages often showing promoting effect on tumor (97). TAMs in malignant tumor usually exhibit an M2-like appearance (98). Macrophages are often regulated by cytokines and other factors, which may promote the occurrence of bone metastases (99). According to the results of current studies, the representative role of different types of immune cells in bone metastases is shown in **Figure 3**. In future studies, we believe that in terms of the immune regulation of bone metastases, how to correctly find the immune cells that promote tumor bone metastases and make them defunction, apoptosis or transform into normal immune cells is the key to the research.

### 3.5 Other mechanisms related to the pathogenesis of bone metastases

**Figure 4:**
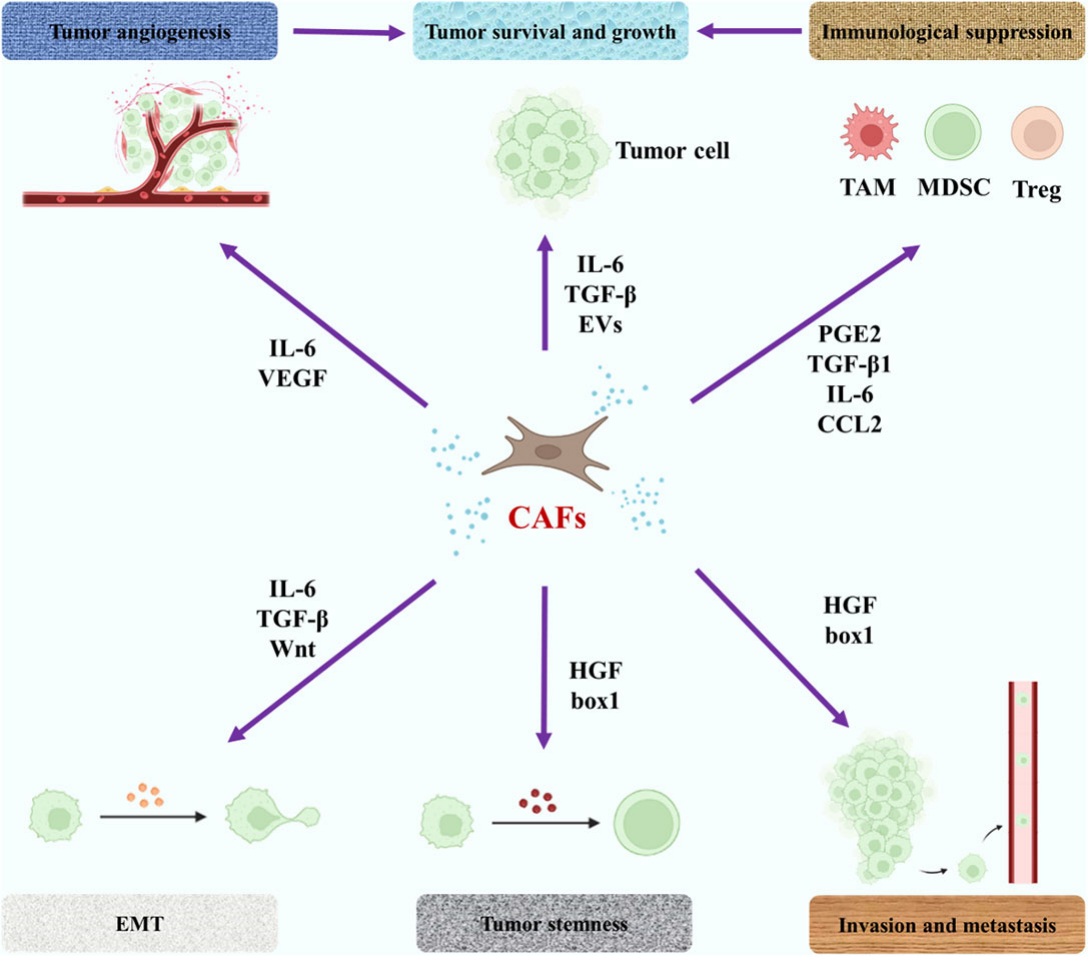
The role of CAFs in the pathogenesis of bone metastases. The purple arrow represents an influence. EMT, epithelial-mesenchymal transition; PGE2, prostaglandin E2; EVs, extracellular vesicles; HGF, hepatocyte growth factor; VEGF, vascular endothelial growth factor.

It can be seen from the above description that the pathogenesis of bone metastases is very complex. As the main function of bone metastases, different types of cells are widely affected by immune, metabolic and tumor microenvironments. In recent years, the role of some connective tissue cells in bone metastases, such as fibroblasts and endothelial cells, in bone metastases has received increasing attention. Similar to macrophages, fibroblasts in malignant tumor tissues are known as cancer-associated fibroblasts (CAFs) and they often show potential tumor-promoting effects (100). CAFs may promote tumor metastasis (101). Li et al. mentioned in their study that CAFs played a key role in bone metastasis of breast cancer cells by influencing tumor microenvironment and other aspects (102). Mukaida et al. fully described the possible effects of CAFs on tumor bone metastasis, including the function of tumor cells and immune cells through the secretion of cytokines by CAFs (103). The relevant content is illustrated in **Figure 4**. In addition to CAFs, the role of endothelial cells in metastases has also been emphasized. Zhang et al. indicated that bone-derived endothelial cells (BDECs) may be involved in pathologic bone lysis in the pathogenesis of bone metastases (104). Wang et al. proposed that tumor cell-vertebral bone marrow endothelial cell interactions promote spinal metastasis in NSCLC (105). In fact, whether in the primary lesion of malignant tumor or the metastasis of bone metastases, tumor cells are only part of the tumor, and the influence of non-tumor cells on the occurrence and development of bone metastases should be paid more attention. Regulation of these cells may have a positive significance in reducing the incidence of bone metastases, delaying the occurrence time of bone metastases, and alleviating the symptoms of bone metastases.

## 4 Clinical treatment prospects of bone metastases

With the deepening of the research on the pathogenesis of bone metastases, clinicians’ understanding of bone metastases is also constantly innovating. In the past, the general consensus reached in clinical practice was that the occurrence of bone metastases in malignant tumors meant that the survival time of patients was shorter, and the treatment should be mainly palliative therapy such as analgesia rather than surgery. However, with the development of scientific progress and clinical research, clinicians gradually found the positive significance of various surgical procedures, especially when there was only a single bone metastasis. With the further study of pathogenesis, some therapies targeting specific cell types are being developed, including advanced nanotechnology therapy. Based on the above scientific research basis, we introduce a series of cutting-edge clinical treatment viewpoints of bone metastases and emerging treatment methods under development.

### 4.1 Progress and prospect of surgical treatment and chemotherapy

The surgical treatment of bone metastases has been paid more attention due to the progress of epidemiological research. In recent years, more emphasis has been placed on the surgical treatment of bone metastases. Surgical treatment of bone metastases often includes pain relief, quality of life improvement and SREs treatment, and may also be used as a means to create conditions for radiotherapy. Because of the complex local pathology, excision may have some positive significance. However, it is important to note that not all patients are candidates for surgery. Whether or not a patient should be treated surgically depends on a number of factors, including systemic conditions, primary tumor status, number and location of metastases, expected survival time, and financial status of the patient. Prior to surgical treatment of bone metastases, it is important to conduct examinations. Some patient-specific scores are important in assessing whether a patient with bone metastases is ready for surgery. For example, for patients with spinal metastases, the Tokuhashi score is a commonly used method to determine whether a patient should be operated on (106). At the same time, the New England Spinal Metastasis Score (NESMS) score had relatively good clinical accuracy in predicting complications after spinal metastasis surgery (107). For patients with limb metastases, Katagiri score might be important references (108, 109). With the development of treatment methods, the surgical methods of spinal metastasis and limb metastasis are gradually diversified. Both open surgery and minimally invasive surgery are used in bone metastases (**Table 2**), and their adaptations have been recognized based on epidemiological studies (115). In particular, the development of new techniques has led to advances in bone metastases surgery. For example, the application of 3D printing technology in joint prostheses enables patients to achieve better motor function and improve the quality of life of patients (116). However, it should be noted that there are potential complications, including intraoperative and postoperative complications, such as spinal cord and vascular injury, failure of internal fixation, local tumor recurrence and so on, no matter what kind of surgery. Bone metastatic tumor surgery has been used as an important treatment method for many patients, but it is different from general orthopaedic trauma surgery, orthopaedic joint surgery and other conventional operations, it is often difficult to operate, high risk, and so far there is a lack of appropriate procedure standards. Therefore, surgery for bone metastases needs to be conducted by an experienced orthopaedic surgeon who carefully evaluates each patient and follows the principle of “personalization.” More epidemiological studies should be carried out.

**Table 2:** Comparison of characteristics of minimally invasive surgery and traditional open surgery.

	Minimally invasive surgery	Traditional open surgery
Type	PVP/PKP (110, 111), RFA (112)	Total vertebrae excision, separation surgery (113, 114)
Complication	Relatively rare	More common, such as wound infection
Blood loss and transfusion rate	The blood loss is small and the transfusion rate is low	Often associated with greater blood loss and higher transfusion rate
Hospital stays	Short	Long

The deepening of scientific research also has a certain impact on the concept of chemotherapy for bone metastases. Surgical treatment of bone metastases usually has limited effects. As mentioned earlier, circulating tumor cells may metastasize before they are detected. Subsequently, these circulating tumor cells may form micrometastases, which are the main cause of tumor recurrence and a major factor affecting survival. Therefore, it is necessary to supplement the corresponding medical treatment. The specific chemotherapy regimen for different bone metastases is different, which is related to the pathological type of the primary lesion.

### 4.2 Targeted therapy—more advanced and promising systemic therapy for bone metastases

With the progress of research on the pathogenesis of bone metastases and primary bone tumors, the concept of “precision therapy” has been gradually formed. Some malignancies may be hormone-related, so some hormone-targeted therapies have been developed, such as Tamoxifen (estrogen inhibitor), Darolutamide (androgen receptor inhibitor), etc. (117, 118) A more widely known type of targeted therapy is targeting specific proteins or signaling pathways, such as Bevacizumab (VEGF inhibitor) (119), Trastuzumab (HER2 inhibitor) (120), Imatinib (tyrosine kinase inhibitor) (121), Olaparib (PARP inhibitor) (122), etc. Other organ-specific drugs such as I^131^ also act as targeted therapies (123). The basic principle of tumor targeted therapy is to design drugs or antibodies for molecules that may be abnormally expressed or have abnormal functions in certain malignant tumors according to epidemiological and pathogenesis studies, so as to interfere with tumor growth and promote tumor killing. Targeted therapy drugs usually cause less damage to normal human tissue than conventional chemotherapy drugs. The combination of targeted therapy with conventional chemotherapy often produces better effects (124). Now, targeted therapy is starting to be used in bone metastases. Tokito et al. showed that bevacizumab may enhance the antitumor activity of chemotherapy against bone metastases and reduce the incidence of SREs (125). A HER2-overexpressed Salivary carcinoma reported by Bergamini et al. developed bone metastases and the treatment plan included trastuzumab (126). It is worth noting that bone-targeting drugs are more widely used in bone metastases. In patients with bone metastases, targeted drugs targeting the primary tumor are often used as a means of comprehensive therapy. In addition to targeting the primary tumor, the more commonly used targeted therapy for bone metastases is “bone-modulatory drugs” for bone lesions, which can be regarded as a type of bone targeting. As mentioned in the previous part, osteoclasts and osteoblasts play an important role in the occurrence of bone metastases. Although the therapeutic effect on tumor is limited, bone targeting drugs can regulate osteoblasts/osteoclasts to inhibit bone destruction and delay the occurrence of SREs, which will greatly improve the quality of life of patients. Some commonly used bone-targeting drugs such as bisphosphonates (BPs) can promote osteoclast apoptosis (127). Denosumab inhibits osteoclast differentiation and activity as a RANK/RANKL inhibitor to delay bone metastasis (128). Some common bone-regulating drugs that inhibit bone destruction in bone metastases are shown in **Table 3**. The positive effect of bone-targeting drugs in bone metastases confirms the necessary for their use in patients.

**Table 3:** Common types of bone regulatory drugs, representative drugs, related mechanisms and typical applications.

Drug class	Representative drug	Mechanism of action	Partial relevant BM treatment	Typical relevant reference
BP	Alendronate, Zoledronate, Risedronate	Inhibits osteoclast activity and promotes osteoclast apoptosis	Pain control/delayed occurrence of SREs in cancer patients with bone metastasis	(129–131)
RANK-L mAb	Denosumab	Inhibits osteoclast differentiation and activity by inhibiting the RANK-RANKL pathway	To reduce the skeletal complications of cancer	(132, 133)
mTOR inhibitor	Everolimus	Inhibition of osteoclast differentiation and activation; Promotion of osteoclast apoptosis	Everolimus plays a bone-protective role in bone metastasis of breast cancer	(134)
Proteasome inhibitor	Bortezomib, Carfilzomib	Inhibits osteoclast formation and promotes osteoblast differentiation	Improves bone destruction in breast cancer	(135)
CYP17 inhibitor	Abiraterone	Inhibits the generation and activity of osteoclasts and promotes the differentiation of osteoblasts	Combined with other BRIs for the treatment of bone metastases from prostate cancer	(136)
Tyrosine kinase inhibitor	Cabozantinib	TKI; Inhibition of VEGF/VEGFR pathway; Regulation of osteoblast activity	Bone metastasis of advanced renal cell carcinoma	(137)
ET-1 antagonist	Bosentan	Regulation of angiogenesis, etc.	–	(138)
DKK-1 inhibitor	–	Promote Wnt pathway and osteoblast differentiation	–	–

However, in practice, targeted drugs are not targeted to tumor cells, which may limit the efficiency of their application to some extent. In recent years, combined with published pathogenesis and clinical studies, more targeted therapies are being developed. Among them, nanotechnology as an emerging means of targeted therapy has attracted wide attention. Nanomaterials can be targeted by a variety of relevant chemical modifications, and properly designed nanomaterials often show high safety and degradability (139). It is a common idea to combine nanotechnology with traditional bone targeting drugs to prepare nanoparticles for the treatment of bone metastases. For example, He et al. have designed a nanoparticle DSP-Zn@PEG-ALN targeted to focal bone *via* the alendronate molecule, which has great potential for improving the efficacy of chemotherapy for bone metastatic breast cancer (140). Qiao et al. highlighted the importance of therapeutic nanomedicine and osteocyte-targeted therapy in the treatment of early bone metastases (141). More representative studies related to nanomaterials with bone targeting drugs for the treatment of bone metastases are presented in **Table 4**. Also, Tamura et al. mentioned in their review that extracellular vesicles may play an important role in tumor bone metastasis, especially in influencing the local tumor microenvironment (148). Ge et al. designed a multifunctional scaffold called CePO4/CS/GO scaffold that promotes bone formation while killing tumors for the treatment of breast cancer with bone metastases (149). These studies are closely related to the pathogenesis of bone metastases. The relevant signaling pathways here have been mentioned in related pathogenesis studies. In future studies, targeting immune cells, osteoblasts or other stromal cells may be an important direction for the innovation of targeted therapy for bone metastases. Until now, the main methods to target nanomaterials to cells have been through specific ligands on the cell surface, through essential substances for cell metabolism, or through the preparation of biomimetic nanoparticles (nanoparticles coated with natural cell membranes, etc.). These methods can be used as reference for the design of nanomaterials for the treatment of bone metastases. The development of new targeted therapies must be strictly dependent on the pathogenesis of bone metastases. Therefore, the development of clinical diagnosis and treatment and the study of pathogenesis are complementary.

**Table 4:** Representative studies related to nanomaterials with bone targeting drugs for the treatment of bone metastases.

Components of bone targeting	The main components of nanomaterials	Loaded components with therapeutic effects	Related disease/models	Reference
Alendronate	PLGA	Curcumin and bortezomib	Breast cancer bone metastasis	(142)
Alendronate	Liposome	Doxorubicin	Breast cancer bone metastasis	(143)
Zoledronic acid	Au@mesoporous silica nanoparticles	Gold nanorods and zoledronic acid	Breast cancer bone metastasis	(144)
cRGD	Complex	Bortezomib	Bone metastasis	(145)
RNA aptamer targeting PSMA	Atelocollagen	miR-15a and miR-16-1	Prostate cancer bone metastasis	(146)
Alendronate and hyaluronic acid	Complex	Doxorubicin	Breast cancer bone metastasis	(147)

## 5 Summary and scope

In summary, we summarized the development status of bone metastases from the aspects of epidemiology, clinical features, pathogenesis and clinical practice. So far, compared with kinds of primary tumors, there are still relatively few researches on bone metastases either in pathogenesis or clinical trials. As the most common malignant tumor of bone, bone metastases should receive more attention in the future. In the future, research on the pathogenesis of bone metastases should focus on the cellular level interaction mechanism. The establishment of animal models of bone metastases is also a very important direction, because successful animal model preparation is the basis of *in vivo* experiments. There should be more studies and reviews on the establishment of *in vivo* models of bone metastases, such as Peng et al. ‘s review of *in vivo* experimental design for intervertebral disc disease (150). Clinical research requires researchers to develop a wide range of new drugs. Nanomaterials are an emerging approach to targeted therapy because they can be multi-functional through modified design. However, its development must rely on the study of pathogenesis, including the discovery of new and effective local targets, how to kill tumors while promoting osteogenesis and tissue recovery, etc. In the future, the comprehensive treatments of bone metastases need to be further improved. The clinician should ensure that the patient has the best quality of life while fully considering the patient’s survival, disease status, and financial status.

## Author contributions

ZS and HH designed the review topic and revised the manuscript. WY and QP conducted data collation and manuscript writing. WY and FH collected data and drew figures. All authors contributed to the article and approved the submitted version.

## References

[1] Coleman RE Croucher PI Padhani AR Clézardin P Chow E Fallon M. Bone metastases. Nat Rev Dis primers (2020) 6(1):83. doi: 10.1016/B978-0-323-47674-4.00056-6 33060614

[2] Ban J Fock V Aryee DNT Kovar H. Mechanisms, diagnosis and treatment of bone metastases. Cells (2021) 10(11):2944. doi: 10.3390/cells10112944 PMC8616226 34831167

[3] Migliorini F Maffulli N Trivellas A Eschweiler J Tingart M Driessen A. Bone metastases: a comprehensive review of the literature. Mol Biol Rep (2020) 47(8):6337–45. doi: 10.1007/s11033-020-05684-0 32749632

[4] Cadieux B Coleman R Jafarinasabian P Lipton A Orlowski RZ Saad F. Experience with denosumab (XGEVA®) for prevention of skeletal-related events in the 10 years after approval. J Bone Oncol (2022) 33:100416. doi: 10.1016/j.jbo.2022.100416 35242510 PMC8857591

[5] Isogai N Yagi M Nishimura S Nishida M Mima Y Hosogane N. Risk predictors of perioperative complications for the palliative surgical treatment of spinal metastasis. J orthopaedic Sci Off J Japanese Orthopaedic Assoc (2021) 26(6):1107–12. doi: 10.1016/j.jos.2020.09.005 34755637

[6] Li C Wu Q Chang D Liang H Ding X Lao C. State-of-the-art of minimally invasive treatments of bone metastases. J Bone Oncol (2022) 34:100425. doi: 10.1016/j.jbo.2022.100425 35391944 PMC8980625

[7] Yang XY Liao JJ Xue WR. FMNL1 down-regulation suppresses bone metastasis through reducing TGF-β1 expression in non-small cell lung cancer (NSCLC). Biomedicine pharmacotherapy = Biomedecine pharmacotherapie (2019) 117:109126. doi: 10.1016/j.biopha.2019.109126 31387165

[8] Liu Z Murphy SF Huang J Zhao L Hall CC Schaeffer AJ. A novel immunocompetent model of metastatic prostate cancer-induced bone pain. Prostate (2020) 80(10):782–94. doi: 10.1002/pros.23993 PMC7375026 32407603

[9] Kondraciuk JD Rice SL Zhou X Gharzeddine K Knezevic A Spratt DE. Thyroid cancer bone metastasis: Survival and genomic characteristics of a Large tertiary care cohort. Clin Nucl Med (2019) 44(8):e465–e71. doi: 10.1097/RLU.0000000000002626 PMC6621602 31274625

[10] Tulotta C Ottewell P. The role of IL-1B in breast cancer bone metastasis. Endocrine-related Cancer (2018) 25(7):R421–r34. doi: 10.1530/ERC-17-0309 PMC5987176 29760166

[11] Haber T Jöckel E Roos FC Junker K Prawitt D Hampel C. Bone metastasis in renal cell carcinoma is preprogrammed in the primary tumor and caused by AKT and integrin α5 signaling. J urology (2015) 194(2):539–46. doi: 10.1016/j.juro.2015.01.079 25623744

[12] [Expert consensus on renal cell carcinoma with bone metastasis (2020 edition)]. Zhonghua zhong liu za zhi [Chinese J oncology] (2020) 42(7):537–42. doi: 10.3760/cma.j.cn112152-20200401-00292 32842439

[13] Wong ECL Kapoor A. Does bone-targeted therapy benefit patients with metastatic renal cell carcinoma? Trans Oncol (2020) 13(2):241–4. doi: 10.1016/j.tranon.2019.10.009 PMC6931200 31869748

[14] Coleman RE. Clinical features of metastatic bone disease and risk of skeletal morbidity. Clin Cancer Res an Off J Am Assoc Cancer Res (2006) 12(20 Pt 2):6243s–9s. doi: 10.1158/1078-0432.CCR-06-0931 17062708

[15] Rath B Tingart M Migliorini F Eschweiler J Zureik R Hardes J. [Differentiated treatment strategies for bone metastases of the extremities]. Der Orthopade (2019) 48(9):752–9. doi: 10.1007/s00132-019-03791-w 31444515

[16] Harel R Angelov L. Spine metastases: current treatments and future directions. Eur J Cancer (Oxford Engl 1990) (2010) 46(15):2696–707. doi: 10.1016/j.ejca.2010.04.025 20627705

[17] McCabe FJ Jadaan MM Byrne F Devitt AT McCabe JP. Spinal metastasis: The rise of minimally invasive surgery. surgeon J R Colleges Surgeons Edinburgh Ireland (2022) 20(5):328–33. doi: 10.1016/j.surge.2021.08.007 34563452

[18] Scutellari PN Antinolfi G Galeotti R Giganti M. [Metastatic bone disease. Strategies imaging] Minerva medica (2003) 94(2):77–90. 12858156

[19] Yildizhan S Boyaci MG Rakip U Aslan A Canbek I. Role of radiofrequency ablation and cement injection for pain control in patients with spinal metastasis. BMC musculoskeletal Disord (2021) 22(1):912. doi: 10.1186/s12891-021-04799-0 PMC8556885 34715849

[20] Guzik G. Oncological and functional results after surgical treatment of bone metastases at the proximal femur. BMC surgery (2018) 18(1):5. doi: 10.1186/s12893-018-0336-0 29370790 PMC5784608

[21] Wedin R Hansen BH Laitinen M Trovik C Zaikova O Bergh P. Complications and survival after surgical treatment of 214 metastatic lesions of the humerus. J shoulder elbow surgery (2012) 21(8):1049–55. doi: 10.1016/j.jse.2011.06.019 21982491

[22] Hashimoto K Ochi H Sunamura S Kosaka N Mabuchi Y Fukuda T. Cancer-secreted hsa-miR-940 induces an osteoblastic phenotype in the bone metastatic microenvironment *via* targeting ARHGAP1 and FAM134A. Proc Natl Acad Sci United States America (2018) 115(9):2204–9. doi: 10.1073/pnas.1717363115 PMC5834702 29440427

[23] Lipton A Uzzo R Amato RJ Ellis GK Hakimian B Roodman GD. The science and practice of bone health in oncology: managing bone loss and metastasis in patients with solid tumors. J Natl Compr Cancer Network JNCCN (2009) 7 Suppl 7(Suppl 7):S1–29. doi: 10.6004/jnccn.2009.0080 PMC3047391 19878635

[24] LeRoy BE Thudi NK Nadella MV Toribio RE Tannehill-Gregg SH van Bokhoven A. New bone formation and osteolysis by a metastatic, highly invasive canine prostate carcinoma xenograft. Prostate (2006) 66(11):1213–22. doi: 10.1002/pros.20408 16683269

[25] LeRoy BE Sellers RS Rosol TJ. Canine prostate stimulates osteoblast function using the endothelin receptors. Prostate (2004) 59(2):148–56. doi: 10.1002/pros.10370 15042615

[26] Wang J Rouse C Jasper JS Pendergast AM. ABL kinases promote breast cancer osteolytic metastasis by modulating tumor-bone interactions through TAZ and STAT5 signaling. Sci Signaling (2016) 9(413):ra12. doi: 10.1126/scisignal.aad3210 PMC4991033 26838548

[27] Quattrocchi CC Piciucchi S Sammarra M Santini D Vincenzi B Tonini G. Bone metastases in breast cancer: Higher prevalence of osteosclerotic lesions. La Radiologia medica (2007) 112(7):1049–59. doi: 10.1007/s11547-007-0205-x 17952675

[28] Pan T Lin SC Yu KJ Yu G Song JH Lewis VO. BIGH3 promotes osteolytic lesions in renal cell carcinoma bone metastasis by inhibiting osteoblast differentiation. Neoplasia (New York NY) (2018) 20(1):32–43. doi: 10.1016/j.neo.2017.11.002 PMC5711998 29190493

[29] Haider MT Ridlmaier N Smit DJ Taipaleenmäki H. Interleukins as mediators of the tumor cell-bone cell crosstalk during the initiation of breast cancer bone metastasis. Int J Mol Sci (2021) 22(6):2898. doi: 10.3390/ijms22062898 PMC7999500 33809315

[30] Otsuka S Hanibuchi M Ikuta K Yano S Goto H Ogino H. A bone metastasis model with osteolytic and osteoblastic properties of human lung cancer ACC-LC-319/bone2 in natural killer cell-depleted severe combined immunodeficient mice. Oncol Res (2009) 17(11-12):581–91. doi: 10.3727/096504009789745511 19806789

[31] Song HJ Wu CG Xue YL Xu YH Qiu ZL Luo QY. Percutaneous osteoplasty combined with radioiodine therapy as a treatment for bone metastasis developing after differentiated thyroid carcinoma. Clin Nucl Med (2012) 37(6):e129–33. doi: 10.1097/RLU.0b013e31824786d0 22614210

[32] Zhang X. Interactions between cancer cells and bone microenvironment promote bone metastasis in prostate cancer. Cancer Commun (London England) (2019) 39(1):76. doi: 10.1186/s40880-019-0425-1 PMC6873445 31753020

[33] Xi X Hu Z Wu Q Hu K Cao Z Zhou J. High expression of small nucleolar RNA host gene 3 predicts poor prognosis and promotes bone metastasis in prostate cancer by activating transforming growth factor-beta signaling. Bioengineered (2022) 13(1):1895–907. doi: 10.1080/21655979.2021.2020393 PMC8805939 35030969

[34] Osorio M Moubayed SP Su H Urken ML. Systematic review of site distribution of bone metastases in differentiated thyroid cancer. Head neck (2017) 39(4):812–8. doi: 10.1002/hed.24655 28079945

[35] Leung AK. Optimizing skeletal-related events prevention in patients with advanced prostate cancer. Asia-Pacific J Clin Oncol (2020) 16 Suppl 3:4–6. doi: 10.1111/ajco.13315 32852902

[36] Bhowmik D Song X Intorcia M Gray S Shi N. Examination of burden of skeletal-related events in patients naive to denosumab and intravenous bisphosphonate therapy in bone metastases from solid tumors population. Curr Med Res opinion (2019) 35(3):513–23. doi: 10.1080/03007995.2018.1532884 30286662

[37] Cao PP Chen WJ Pang HL Shen WW Xue P Duan L. Effect of CUL4A on the metastatic potential of lung adenocarcinoma to the bone. Oncol Rep (2020) 43(2):662–70. doi: 10.3892/or.2019.7448 31894344

[38] Miyashita H Cruz C Patel V. Risk factors of skeletal-related events in patients with bone metastatic castration-resistant prostate cancer undergoing treatment with zoledronate. Supportive Care Cancer Off J Multinational Assoc Supportive Care Cancer (2022) 30(2):981–4. doi: 10.1007/s00520-021-06340-4 34373957

[39] Yu L Sui B Fan w Lei L Zhou L Yang L. Exosomes derived from osteogenic tumor activate osteoclast differentiation and concurrently inhibit osteogenesis by transferring COL1A1-targeting miRNA-92a-1-5p. J Extracell Vesicles. (2021) 10(3):e12056. doi: 10.1002/jev2.12056 PMC7812369 33489015

[40] Navarro-Gonzalez E. Use of multikinase inhibitors/lenvatinib concomitant with antiresorptive therapy for bone metastases from radioiodine-resistant differentiated thyroid cancer. Cancer Med (2022) 11 Suppl 1(Suppl 1):54–8. doi: 10.1002/cam4.4983 PMC9537057 35785524

[41] Valastyan S Weinberg RA. Tumor metastasis: Molecular insights and evolving paradigms. Cell (2011) 147(2):275–92. doi: 10.1016/j.cell.2011.09.024 PMC3261217 22000009

[42] Clézardin P Coleman R Puppo M Ottewell P Bonnelye E Paycha F. Bone Metastasis: mechanisms, therapies, and biomarkers. Physiol Rev (2021) 101(3):797–855. doi: 10.1152/physrev.00012.2019 33356915

[43] Huang X Shi X Huang D Li B Lin N Pan W. Mutational characteristics of bone metastasis of lung cancer. Ann palliative Med (2021) 10(8):8818–26. doi: 10.21037/apm-21-1595 34488370

[44] Arnold RS Fedewa SA Goodman M Osunkoya AO Kissick HT Morrissey C. Bone metastasis in prostate cancer: Recurring mitochondrial DNA mutation reveals selective pressure exerted by the bone microenvironment. Bone (2015) 78:81–6. doi: 10.1016/j.bone.2015.04.046 PMC4466124 25952970

[45] Bartels S Christgen M Luft A Persing S Jödecke K Lehmann U. Estrogen receptor (ESR1) mutation in bone metastases from breast cancer. Modern Pathol an Off J United States Can Acad Pathology Inc (2018) 31(1):56–61. doi: 10.1038/modpathol.2017.95 28799536

[46] Yilmaz M Christofori G. EMT, the cytoskeleton, and cancer cell invasion. Cancer metastasis Rev (2009) 28(1-2):15–33. doi: 10.1007/s10555-008-9169-0 19169796

[47] Lamouille S Xu J Derynck R. Molecular mechanisms of epithelial-mesenchymal transition. Nat Rev Mol Cell Biol (2014) 15(3):178–96. doi: 10.1038/nrm3758 PMC4240281 24556840

[48] Liu L Chen X Wang Y Qu Z Lu Q Zhao J. Notch3 is important for TGF-β-induced epithelial-mesenchymal transition in non-small cell lung cancer bone metastasis by regulating ZEB-1. Cancer Gene Ther (2014) 21(9):364–72. doi: 10.1038/cgt.2014.39 25080992

[49] Horas K Zheng Y Fong-Yee C Macfarlane E Manibo J Chen Y. Loss of the vitamin d receptor in human breast cancer cells promotes epithelial to mesenchymal cell transition and skeletal colonization. J Bone mineral Res Off J Am Soc Bone Mineral Res (2019) 34(9):1721–32. doi: 10.1002/jbmr.3744 30995345

[50] Huang S Wa Q Pan J Peng X Ren D Huang Y. Downregulation of miR-141-3p promotes bone metastasis *via* activating NF-κB signaling in prostate cancer. J Exp Clin Cancer Res CR (2017) 36(1):173. doi: 10.1186/s13046-017-0645-7 29202848 PMC5716366

[51] Ren D Yang Q Dai Y Guo W Du H Song L. Oncogenic miR-210-3p promotes prostate cancer cell EMT and bone metastasis *via* NF-κB signaling pathway. Mol cancer (2017) 16(1):117 doi: 10.1186/s12943-017-0688-6. 28693582 PMC5504657

[52] Bergers G Fendt SM. The metabolism of cancer cells during metastasis. Nat Rev Cancer (2021) 21(3):162–80. doi: 10.1038/s41568-020-00320-2 PMC8733955 33462499

[53] Nimmakayala RK Leon F Rachagani S Rauth S Nallasamy P Marimuthu S. Metabolic programming of distinct cancer stem cells promotes metastasis of pancreatic ductal adenocarcinoma. Oncogene (2021) 40(1):215–31. doi: 10.1038/s41388-020-01518-2 PMC10041665 33110235

[54] Thysell E Surowiec I Hörnberg E Crnalic S Widmark A Johansson AI. Metabolomic characterization of human prostate cancer bone metastases reveals increased levels of cholesterol. PloS One (2010) 5(12):e14175. doi: 10.1371/journal.pone.0014175 21151972 PMC2997052

[55] Luo A Xu Y Li S Bao J Lü J Ding N. Cancer stem cell property and gene signature in bone-metastatic breast cancer cells. Int J Biol Sci (2020) 16(14):2580–94. doi: 10.7150/ijbs.45693 PMC7415422 32792858

[56] Huang J Freyhult E Buckland R Josefsson A Damber JE Welén K. Osteoclasts directly influence castration-resistant prostate cancer cells. Clin Exp metastasis (2022) 39(5):801–14. doi: 10.1007/s10585-022-10179-2 PMC9474581 35971022

[57] Yang H Yu Z Ji S Huo Q Yan J Gao Y. Targeting bone microenvironments for treatment and early detection of cancer bone metastatic niches. J Controlled release Off J Controlled Release Society (2022) 341:443–56. doi: 10.1016/j.jconrel.2021.11.005 34748870

[58] Kähkönen TE Halleen JM Bernoulli J. Osteoimmuno-oncology: Therapeutic opportunities for targeting immune cells in bone metastasis. Cells (2021) 10(6):1529. doi: 10.3390/cells10061529 PMC8233913 34204474

[59] Liu SC Sun QZ Qiao XF Li XG Yang JH Wang TQ. LncRNA TUG1 influences osteoblast proliferation and differentiation through the wnt/β-catenin signaling pathway. Eur Rev Med Pharmacol Sci (2019) 23(11):4584–90. doi: 10.26355/eurrev_201906_18035 31210284

[60] Udagawa N Koide M Nakamura M Nakamichi Y Yamashita T Uehara S. Osteoclast differentiation by RANKL and OPG signaling pathways. J Bone mineral Metab (2021) 39(1):19–26. doi: 10.1007/s00774-020-01162-6 33079279

[61] Liu XH Kirschenbaum A Weinstein BM Zaidi M Yao S Levine AC. Prostaglandin E2 modulates components of the wnt signaling system in bone and prostate cancer cells. Biochem Biophys Res Commun (2010) 394(3):715–20. doi: 10.1016/j.bbrc.2010.03.057 20227393

[62] Dai J Hall CL Escara-Wilke J Mizokami A Keller JM Keller ET. Prostate cancer induces bone metastasis through wnt-induced bone morphogenetic protein-dependent and independent mechanisms. Cancer Res (2008) 68(14):5785–94. doi: 10.1158/0008-5472.CAN-07-6541 PMC4432935 18632632

[63] Aufderklamm S Hennenlotter J Leidenberger P Rausch S Hohneder A Kühs U. Systemic alterations of wnt inhibitors in patients with prostate cancer and bone metastases. Dis markers (2018) 2018:1874598. doi: 10.1155/2018/1874598 30116403 PMC6079590

[64] Takayama K Inoue T Narita S Maita S Huang M Numakura K. Inhibition of the RANK/RANKL signaling with osteoprotegerin prevents castration-induced acceleration of bone metastasis in castration-insensitive prostate cancer. Cancer letters (2017) 397:103–10. doi: 10.1016/j.canlet.2017.03.034 28373003

[65] Kopper L. Denosumab–a powerful RANKL inhibitor to stop lytic metastases and other bone loss actions by osteoclasts. Pathol Oncol Res POR (2012) 18(4):743–7. doi: 10.1007/s12253-012-9538-4 22588706

[66] Li B Wang P Jiao J Wei H Xu W Zhou P. Roles of the RANKL-RANK axis in immunity-implications for pathogenesis and treatment of bone metastasis. Front Immunol (2022) 13:824117. doi: 10.3389/fimmu.2022.824117 35386705 PMC8977491

[67] Chen YC Sosnoski DM Mastro AM. Breast cancer metastasis to the bone: Mechanisms of bone loss. Breast Cancer Res BCR (2010) 12(6):215. doi: 10.1186/bcr2781 21176175 PMC3046443

[68] Chauhan P Nair A Patidar A Dandapat J Sarkar A. Saha b. A primer cytokines Cytokine (2021) 145:155458. doi: 10.1016/j.cyto.2021.155458 33581983

[69] Propper DJ Balkwill FR. Harnessing cytokines and chemokines for cancer therapy. Nat Rev Clin Oncol (2022) 19(4):237–53. doi: 10.1038/s41571-021-00588-9 34997230

[70] Harmer D Falank C Reagan MR. Interleukin-6 interweaves the bone marrow microenvironment, bone loss, and multiple myeloma. Front endocrinology (2018) 9:788. doi: 10.3389/fendo.2018.00788 PMC6333051 30671025

[71] He Y Luo W Liu Y Wang Y Ma C Wu Q. IL-20RB mediates tumoral response to osteoclastic niches and promotes bone metastasis of lung cancer. J Clin Invest (2022) 132(20):e157917. doi: 10.1172/JCI157917 36006737 PMC9566910

[72] Hamaguchi T Wakabayashi H Matsumine A Sudo A Uchida A. TNF inhibitor suppresses bone metastasis in a breast cancer cell line. Biochem Biophys Res Commun (2011) 407(3):525–30. doi: 10.1016/j.bbrc.2011.03.051 21414299

[73] Lee JS Lee JB Cha JK Choi EY Park SY Cho KS. Chemokine in inflamed periodontal tissues activates healthy periodontal-ligament stem cell migration. J Clin periodontology (2017) 44(5):530–9. doi: 10.1111/jcpe.12710 28207939

[74] Weidle UH Birzele F Kollmorgen G Rüger R. Molecular mechanisms of bone metastasis. Cancer Genomics proteomics (2016) 13(1):1–12. 26708594

[75] Guérard A Laurent V Fromont G Estève D Gilhodes J Bonnelye E. The chemokine receptor CCR3 is potentially involved in the homing of prostate cancer cells to bone: Implication of bone-marrow adipocytes. Int J Mol Sci (2021) 22(4):1994. doi: 10.3390/ijms22041994 PMC7922974 33671469

[76] Zitka O Kukacka J Krizkova S Huska D Adam V Masarik M. Matrix metalloproteinases. Curr medicinal Chem (2010) 17(31):3751–68. doi: 10.2174/092986710793213724 20846107

[77] Pego ER Fernández I Núñez MJ. Molecular basis of the effect of MMP-9 on the prostate bone metastasis: A review. Urologic Oncol (2018) 36(6):272–82. doi: 10.1016/j.urolonc.2018.03.009 29650324

[78] Zhang X Yu X Zhao Z Yuan Z Ma P Ye Z. MicroRNA-429 inhibits bone metastasis in breast cancer by regulating CrkL and MMP-9. Bone (2020) 130:115139. doi: 10.1016/j.bone.2019.115139 31706051

[79] Morrison C Mancini S Cipollone J Kappelhoff R Roskelley C Overall C. Microarray and proteomic analysis of breast cancer cell and osteoblast co-cultures: role of osteoblast matrix metalloproteinase (MMP)-13 in bone metastasis. J Biol Chem (2011) 286(39):34271–85. doi: 10.1074/jbc.M111.222513 PMC3190775 21784845

[80] Frejborg E Salo T Salem A. Role of cyclooxygenase-2 in head and neck tumorigenesis. Int J Mol Sci (2020) 21(23):9246. doi: 10.3390/ijms21239246 PMC7731111 33287464

[81] Li Z Schem C Shi YH Medina D Zhang M. Increased COX2 expression enhances tumor-induced osteoclastic lesions in breast cancer bone metastasis. Clin Exp metastasis (2008) 25(4):389–400. doi: 10.1007/s10585-007-9117-3 17965942

[82] Karavitis J Zhang M. COX2 regulation of breast cancer bone metastasis. Oncoimmunology (2013) 2(3):e23129. doi: 10.4161/onci.23129 23802065 PMC3661150

[83] Zhang J Pang Y Xie T Zhu L. CXCR4 antagonism in combination with IDO1 inhibition weakens immune suppression and inhibits tumor growth in mouse breast cancer bone metastases. OncoTargets Ther (2019) 12:4985–92. doi: 10.2147/OTT.S200643 PMC6607200 31388305

[84] Okamoto K Takayanagi H. Osteoimmunology. Cold Spring Harbor Perspect Med (2019) 9(1):a031245. doi: 10.1101/cshperspect.a031245 PMC6314075 29610150

[85] Fernández Vallone VB Hofer EL Choi H Bordenave RH Batagelj E Feldman L. Behaviour of mesenchymal stem cells from bone marrow of untreated advanced breast and lung cancer patients without bone osteolytic metastasis. Clin Exp metastasis (2013) 30(3):317–32. doi: 10.1007/s10585-012-9539-4 23053744

[86] Wu MY Li CJ Yiang GT Cheng YL Tsai AP Hou YT. Molecular regulation of bone metastasis pathogenesis. Cell Physiol Biochem Int J Exp Cell physiology biochemistry Pharmacol (2018) 46(4):1423–38. doi: 10.1159/000489184 29689559

[87] Zhao Y Chen W Zhu W Meng H Chen J Zhang J. Overexpression of interferon regulatory factor 7 (IRF7) reduces bone metastasis of prostate cancer cells in mice. Oncol Res (2017) 25(4):511–22. doi: 10.3727/096504016X14756226781802 PMC7841009 27733217

[88] Bidwell BN Slaney CY Withana NP Forster S Cao Y Loi S. Silencing of Irf7 pathways in breast cancer cells promotes bone metastasis through immune escape. Nat Med (2012) 18(8):1224–31. doi: 10.1038/nm.2830 22820642

[89] Zou W. Regulatory T cells, tumour immunity and immunotherapy. Nat Rev Immunol (2006) 6(4):295–307. doi: 10.1038/nri1806 16557261

[90] Zou L Barnett B Safah H Larussa VF Evdemon-Hogan M Mottram P. Bone marrow is a reservoir for CD4+CD25+ regulatory T cells that traffic through CXCL12/CXCR4 signals. Cancer Res (2004) 64(22):8451–5. doi: 10.1158/0008-5472.CAN-04-1987 15548717

[91] Tan W Zhang W Strasner A Grivennikov S Cheng JQ Hoffman RM. Tumour-infiltrating regulatory T cells stimulate mammary cancer metastasis through RANKL-RANK signalling. Nature (2011) 470(7335):548–53. doi: 10.1038/nature09707 PMC3166217 21326202

[92] Jarnicki AG Lysaght J Todryk S Mills KH. Suppression of antitumor Immunity by IL-10 and TGF-beta-producing T cells infiltrating the growing tumor: influence of tumor environment on the induction of CD4+ and CD8+ regulatory T cells. J Immunol (Baltimore Md 1950) (2006) 177(2):896–904. doi: 10.4049/jimmunol.177.2.896 16818744

[93] Collison LW Workman CJ Kuo TT Boyd K Wang Y Vignali KM. The inhibitory cytokine IL-35 contributes to regulatory T-cell function. Nature (2007) 450(7169):566–9. doi: 10.1038/nature06306 18033300

[94] Muscarella AM Aguirre S Hao X Waldvogel SM Zhang XH. Exploiting bone niches: progression of disseminated tumor cells to metastasis. J Clin Invest (2021) 131(6):e143764. doi: 10.1172/JCI143764 PMC7954594 33720051

[95] Consonni FM Bleve A Totaro MG Storto M Kunderfranco P Termanini A. Heme catabolism by tumor-associated macrophages controls metastasis formation. Nat Immunol (2021) 22(5):595–606. doi: 10.1038/s41590-021-00921-5 33903766

[96] Ye Y Xu Y Lai Y He W Li Y Wang R. Long non-coding RNA cox-2 prevents immune evasion and metastasis of hepatocellular carcinoma by altering M1/M2 macrophage polarization. J Cell Biochem (2018) 119(3):2951–63. doi: 10.1002/jcb.26509 29131381

[97] Zhao CC Han QJ Ying HY Gu XX Yang N Li LY. TNFSF15 facilitates differentiation and polarization of macrophages toward M1 phenotype to inhibit tumor growth. Oncoimmunology (2022) 11(1):2032918. doi: 10.1080/2162402X.2022.2032918 35127254 PMC8812784

[98] Tariq M Zhang JQ Liang GK He QJ Ding L Yang B. Gefitinib inhibits M2-like polarization of tumor-associated macrophages in Lewis lung cancer by targeting the STAT6 signaling pathway. Acta pharmacologica Sinica (2017) 38(11):1501–11. doi: 10.1038/aps.2017.124 PMC5672074 29022575

[99] Ma RY Zhang H Li XF Zhang CB Selli C Tagliavini G. Monocyte-derived macrophages promote breast cancer bone metastasis outgrowth. . J Exp Med (2020) 217(11):e20191820. doi: 10.1084/jem.20191820 32780802 PMC7596825

[100] Lin Y Cai Q Chen Y Shi T Liu W Mao L. CAFs shape myeloid-derived suppressor cells to promote stemness of intrahepatic cholangiocarcinoma through 5-lipoxygenase. Hepatol (Baltimore Md) (2022) 75(1):28–42. doi: 10.1002/hep.32099 34387870

[101] Paauwe M Schoonderwoerd MJA Helderman R Harryvan TJ Groenewoud A van Pelt GW. Endoglin expression on cancer-associated fibroblasts regulates invasion and stimulates colorectal cancer metastasis. Clin Cancer Res an Off J Am Assoc Cancer Res (2018) 24(24):6331–44. doi: 10.1158/1078-0432.CCR-18-0329 29945992

[102] Li H Lin X Yang D Chen Z Wang X Re F. Cancer-associated fibroblasts support bone tropic metastasis by acting as coordinators between the tumor microenvironment and bone matrix in breast cancer. Neoplasma (2021) 68(1):10–22. doi: 10.4149/neo_2020_200905N951 33231088

[103] Mukaida N Zhang D Sasaki SI. Emergence of cancer-associated fibroblasts as an indispensable cellular player in bone metastasis process. Cancers (2020) 12(10):2896. doi: 10.3390/cancers12102896 PMC7600711 33050237

[104] Zhang Y Fujita N Oh-hara T Morinaga Y Nakagawa T Yamada M. Production of interleukin-11 in bone-derived endothelial cells and its role in the formation of osteolytic bone metastasis. Oncogene (1998) 16(6):693–703. doi: 10.1038/sj.onc.1201581 9488033

[105] Wang K Jiang L Hu A Sun C Zhou L Huang Y. Vertebral-specific activation of the CX3CL1/ICAM-1 signaling network mediates non-small-cell lung cancer spinal metastasis by engaging tumor cell-vertebral bone marrow endothelial cell interactions. Theranostics (2021) 11(10):4770–89. doi: 10.7150/thno.54235 PMC7978319 33754027

[106] Eap C Tardieux E Goasgen O Bennis S Mireau E Delalande B. Tokuhashi score and other prognostic factors in 260 patients with surgery for vertebral metastases. Orthopaedics traumatology Surg Res OTSR (2015) 101(4):483–8. doi: 10.1016/j.otsr.2015.03.007 25910701

[107] De la Garza Ramos R Naidu I Choi JH Pennington Z Goodwin CR Sciubba DM. Comparison of three predictive scoring systems for morbidity in oncological spine surgery. J Clin Neurosci Off J Neurosurgical Soc Australasia (2021) 94:13–7. doi: 10.1016/j.jocn.2021.09.031 34863427

[108] Tanaka A Katagiri H Murata H Wasa J Miyagi M Honda Y. Surgery for femoral metastases. Bone Joint J (2020) 102-b(3):285–92. doi: 10.1302/0301-620X.102B3.BJJ-2019-0976.R1 32114815

[109] Hoban H Soejima T Sulaiman NS Sekii S Matsumoto Y Ota Y. Predicting the survival of patients with bone metastases treated with radiation therapy: A validation study of the Katagiri scoring system? Radiat Oncol. (2019) 14(1):13. doi: 10.1186/s13014-019-1218-z PMC6339356 30658673

[110] Sun G Li L Jin P Liu XW Li M. Percutaneous vertebroplasty for painful spinal metastasis with epidural encroachment. J Surg Oncol (2014) 110(2):123–8. doi: 10.1002/jso.23608 24665071

[111] Chen F Xia YH Cao WZ Shan W Gao Y Feng BO. Percutaneous kyphoplasty for the treatment of spinal metastases. Oncol letters (2016) 11(3):1799–806. doi: 10.3892/ol.2016.4121 PMC4774488 26998079

[112] Sayed D Jacobs D Sowder T Haines D Orr W. Spinal radiofrequency ablation combined with cement augmentation for painful spinal vertebral metastasis: A single-center prospective study. Pain physician (2019) 22(5):E441–e9. doi: 10.36076/ppj/2019.22.E441 31561656

[113] Li RF Qiao RQ Xu MY Ma RX Hu YC. Separation surgery in the treatment of spinal metastasis. Technol Cancer Res Treat (2022) 21:15330338221107208. doi: 10.1177/15330338221107208 35702739 PMC9208034

[114] Liu DD Leary OP Camara-Quintana JQ Galgano MA. Two-Level Separation Surgery for Thoracic Epidural Metastatic Disease: An Operative Video Demonstration. World Neurosurg. (2021) 147::160. doi: 10.1016/j.wneu.2020.12.022 33316479

[115] Pranata R Lim MA Vania R Bagus Mahadewa TG. Minimal invasive surgery instrumented fusion versus conventional open surgical instrumented fusion for the treatment of spinal metastases: A systematic review and meta-analysis. World neurosurgery (2021) 148:e264–e74. doi: 10.1016/j.wneu.2020.12.130 33418123

[116] Fang X Lei S Luo Y Zhou Y Min L Zhang W. Research on three-dimensional printing technology based on three-dimensional multimodality imaging to assist the operation of malignant bone tumors of limbs. Zhongguo xiu fu chong jian wai ke za zhi = Zhongguo xiufu chongjian waike zazhi = Chin J reparative reconstructive surgery (2022) 36(7):804–10. doi: 10.7507/1002-1892.202202060 PMC9288904 35848174

[117] Osborne CK. Tamoxifen in the treatment of breast cancer. New Engl J Med (1998) 339(22):1609–18. doi: 10.1056/NEJM199811263392207 9828250

[118] Smith MR Hussain M Saad F Fizazi K Sternberg CN Crawford ED. Darolutamide and survival in metastatic, hormone-sensitive prostate cancer. New Engl J Med (2022) 386(12):1132–42. doi: 10.1056/NEJMoa2119115 PMC9844551 35179323

[119] Garcia J Hurwitz HI Sandler AB Miles D Coleman RL Deurloo R. Bevacizumab (Avastin®) in cancer treatment: A review of 15 years of clinical experience and future outlook. Cancer Treat Rev (2020) 86:102017. doi: 10.1016/j.ctrv.2020.102017 32335505

[120] Watanabe S Yonesaka K Tanizaki J Nonagase Y Takegawa N Haratani k. Targeting of the HER2/HER3 signaling axis overcomes ligand-mediated resistance to trastuzumab in HER2-positive breast cancer. Cancer Med. (2019) 8(3):1258–1268. doi: 10.1002/cam4.1995 PMC6434202 30701699

[121] Kang J Lee JY Park JH Chang DJ. Synthesis of imatinib, a tyrosine kinase inhibitor, labeled with carbon-14. J labelled compounds radiopharmaceuticals (2020) 63(4):174–82. doi: 10.1002/jlcr.3830 31975483

[122] de Bono J Mateo J Fizazi K Saad F Shore N Sandhu S. Olaparib for metastatic castration-resistant prostate cancer. New Engl J Med (2020) 382(22):2091–102. doi: 10.1056/NEJMoa1911440 32343890

[123] Chung JK Cheon GJ. Radioiodine therapy in differentiated thyroid cancer: the first targeted therapy in oncology. Endocrinol Metab (Seoul). (2014) 29(3):233–9. doi: 10.3803/EnM.2014.29.3.233 PMC4192819 25309780

[124] Rodler ET Kurland BF Griffin M Gralow JR Porter P Yeh RF. Phase I study of veliparib (ABT-888) combined with cisplatin and vinorelbine in advanced triple-negative breast cancer and/or BRCA mutation-associated breast cancer. Clin Cancer Res an Off J Am Assoc Cancer Res (2016) 22(12):2855–64. doi: 10.1158/1078-0432.CCR-15-2137 PMC4911292 26801247

[125] Tokito T Shukuya T Akamatsu H Taira T Ono A Kenmotsu H. Efficacy of bevacizumab-containing chemotherapy for non-squamous non-small cell lung cancer with bone metastases. Cancer chemotherapy Pharmacol (2013) 71(6):1493–8. doi: 10.1007/s00280-013-2148-3 23532208

[126] Bergamini C Cavalieri S Sanguineti G Farneti A Licitra L. Treatment of HER2+ metastatic salivary ductal carcinoma in a pregnant woman: a case report. Oxford Med Case Rep (2019) 2019(10):omz102. doi: 10.1093/omcr/omz102 PMC6822604 31772741

[127] Singh T Kaur V Kumar M Kaur P Murthy RS Rawal RK. The critical role of bisphosphonates to target bone cancer metastasis: an overview. J Drug targeting (2015) 23(1):1–15. doi: 10.3109/1061186X.2014.950668 25203856

[128] Smith MR Saad F Coleman R Shore N Fizazi K Tombal B. Denosumab and bone-metastasis-free survival in men with castration-resistant prostate cancer: results of a phase 3, randomised, placebo-controlled trial. Lancet (London England) (9810) 2012:39– 46:379. doi: 10.1016/S0140-6736(11)61226-9 PMC3671878 22093187

[129] Goldvaser H Amir E. Role of bisphosphonates in breast cancer therapy. Curr Treat options Oncol (2019) 20(4):26. doi: 10.1007/s11864-019-0623-8 30874905

[130] Hosaka S Katagiri H Niwakawa M Harada H Wasa J Murata H. Radiotherapy combined with zoledronate can reduce skeletal-related events in renal cell carcinoma patients with bone metastasis. Int J Clin Oncol (2018) 23(6):1127–33. doi: 10.1007/s10147-018-1310-7 29959563

[131] Wei Z Pan B Jia D Yu Y. Long-term safety and efficacy of bisphosphonate therapy in advanced lung cancer with bone metastasis. Future Oncol (London England) (2022) 18(18):2257–67. doi: 10.2217/fon-2022-0098 35414201

[132] Chen SC Kuo PL. Bone metastasis from renal cell carcinoma. Int J Mol Sci (2016) 17(6):987. doi: 10.3390/ijms17060987 PMC4926516 27338367

[133] Irelli A Sirufo MM D'Ugo C Ginaldi L De Martinis M. Real-life use of denosumab 120 mg every 12 weeks in prolonged treatment over 2 years of patients with breast cancer bone metastases. J BUON Off J Balkan Union Oncol (2020) 25(4):1799–804. 33099916

[134] Browne AJ Kubasch ML Göbel A Hadji P Chen D Rauner M. Concurrent antitumor and bone-protective effects of everolimus in osteotropic breast cancer. Breast Cancer Res BCR (2017) 19(1):92. doi: 10.1186/s13058-017-0885-7 28793923 PMC5551016

[135] Jones MD Liu JC Barthel TK Hussain S Lovria E Cheng D. A proteasome inhibitor, bortezomib, inhibits breast cancer growth and reduces osteolysis by downregulating metastatic genes. Clin Cancer Res an Off J Am Assoc Cancer Res (2010) 16(20):4978–89. doi: 10.1158/1078-0432.CCR-09-3293 PMC2955762 20843837

[136] Francini E Montagnani F Nuzzo PV Gonzalez-Velez M Alimohamed NS Rosellini P. Association of concomitant bone resorption inhibitors with overall survival among patients with metastatic castration-resistant prostate cancer and bone metastases receiving abiraterone acetate with prednisone as first-line therapy. JAMA network Open (2021) 4(7):e2116536. doi: 10.1001/jamanetworkopen.2021.16536 34292336 PMC8299314

[137] Escudier B Powles T Motzer RJ Olencki T Arén Frontera O Oudard S. Cabozantinib, a new standard of care for patients with advanced renal cell carcinoma and bone metastases? subgroup analysis of the METEOR trial. J Clin Oncol Off J Am Soc Clin Oncol (2018) 36(8):765–72. doi: 10.1200/JCO.2017.74.7352 PMC6804840 29309249

[138] Dréau D Karaa A Culberson C Wyan H McKillop IH Clemens MG. Bosentan inhibits tumor vascularization and bone metastasis in an immunocompetent skin-fold chamber model of breast carcinoma cell metastasis. Clin Exp metastasis (2006) 23(1):41–53. doi: 10.1007/s10585-006-9016-z 16826430

[139] Tan R Wan Y Yang X. Hydroxyethyl starch and its derivatives as nanocarriers for delivery of diagnostic and therapeutic agents towards cancers. Biomaterials translational (2020) 1(1):46–57. doi: 10.3877/cma.j.issn.2096-112X.2020.01.005 35837654 PMC9255820

[140] He Y Huang Y Huang Z Jiang Y Sun X Shen Y. Bisphosphonate-functionalized coordination polymer nanoparticles for the treatment of bone metastatic breast cancer. J Controlled release Off J Controlled Release Society (2017) 264:76–88. doi: 10.1016/j.jconrel.2017.08.024 28842315

[141] Qiao H Cui Z Yang S Ji D Wang Y Yang Y. Targeting osteocytes to attenuate early breast cancer bone metastasis by theranostic upconversion nanoparticles with responsive plumbagin release. ACS nano (2017) 11(7):7259–73. doi: 10.1021/acsnano.7b03197 28692257

[142] Thamake SI Raut SL Gryczynski Z Ranjan AP Vishwanatha JK. Alendronate coated poly-lactic-co-glycolic acid (PLGA) nanoparticles for active targeting of metastatic breast cancer. Biomaterials (2012) 33(29):7164–73. doi: 10.1016/j.biomaterials.2012.06.026 22795543

[143] Dos Santos Ferreira D Jesus de Oliveira Pinto BL Kumar V Cardoso VN Fernandes SO Souza CM. Evaluation of antitumor activity and cardiac toxicity of a bone-targeted ph-sensitive liposomal formulation in a bone metastasis tumor model in mice. Nanomedicine nanotechnology biology Med (2017) 13(5):1693–701. doi: 10.1016/j.nano.2017.03.005 PMC5483199 28343016

[144] Sun W Ge K Jin Y Han Y Zhang H Zhou G. Bone-targeted nanoplatform combining zoledronate and photothermal therapy to treat breast cancer bone metastasis. ACS nano (2019) 13(7):7556–67. doi: 10.1021/acsnano.9b00097 31259530

[145] Wang M Cai X Yang J Wang C Tong L Xiao J. A targeted and pH-responsive bortezomib nanomedicine in the treatment of metastatic bone tumors. ACS Appl materials interfaces (2018) 10(48):41003–11. doi: 10.1021/acsami.8b07527 30403331

[146] Hao Z Fan W Hao J Wu X Zeng GQ Zhang LJ. Efficient delivery of micro RNA to bone-metastatic prostate tumors by using aptamer-conjugated atelocollagen *in vitro* and in vivo. Drug delivery (2016) 23(3):874–81. doi: 10.3109/10717544.2014.9200597 24892627

[147] Zhao YP Ye WL Liu DZ Cui H Cheng Y Liu M. Redox and pH dual sensitive bone targeting nanoparticles to treat breast cancer bone metastases and inhibit bone resorption. Nanoscale (2017) 9(19):6264–77. doi: 10.1039/C7NR00962C 28470315

[148] Tamura T Yoshioka Y Sakamoto S Ichikawa T Ochiya T. Extracellular vesicles in bone metastasis: Key players in the tumor microenvironment and promising therapeutic targets. Int J Mol Sci (2020) 21(18):6680. doi: 10.3390/ijms21186680 PMC7555648 32932657

[149] Ge YW Liu XL Yu DG Zhu ZA Ke QF Mao YQ. Graphene-modified CePO4 nanorods effectively treat breast cancer-induced bone metastases and regulate macrophage polarization to improve osteo-inductive ability. J nanobiotechnology (2021) 19(1):11. doi: 10.1186/s12951-020-00753-9 33413447 PMC7792230

[150] Peng Y Qing X Shu H Tian S Yang W Chen S. Proper animal experimental designs for preclinical research of biomaterials for intervertebral disc regeneration. Biomaterials translational (2021) 2(2):91–142. doi: 10.12336/biomatertransl.2021.02.003 35836965 PMC9255780

